# Protein B5 is required on extracellular enveloped vaccinia virus for repulsion of superinfecting virions

**DOI:** 10.1099/vir.0.043943-0

**Published:** 2012-09

**Authors:** Virginie Doceul, Michael Hollinshead, Adrien Breiman, Kathlyn Laval, Geoffrey L. Smith

**Affiliations:** Section of Virology, Division of Infectious Diseases, Faculty of Medicine, Imperial College London, St Mary’s campus, Norfolk Place, London W2 1PG, UK

## Abstract

Vaccinia virus (VACV) spreads across cell monolayers fourfold faster than predicted from its replication kinetics. Early after infection, infected cells repulse some superinfecting extracellular enveloped virus (EEV) particles by the formation of actin tails from the cell surface, thereby causing accelerated spread to uninfected cells. This strategy requires the expression of two viral proteins, A33 and A36, on the surface of infected cells and upon contact with EEV this complex induces actin polymerization. Here we have studied this phenomenon further and investigated whether A33 and A36 expression in cell lines causes an increase in VACV plaque size, whether these proteins are able to block superinfection by EEV, and which protein(s) on the EEV surface are required to initiate the formation of actin tails from infected cells. Data presented show that VACV plaque size was not increased by expression of A33 and A36, and these proteins did not block entry of the majority of EEV binding to these cells. In contrast, expression of proteins A56 and K2 inhibited entry of both EEV and intracellular mature virus. Lastly, VACV protein B5 was required on EEV to induce the formation of actin tails at the surface of cells expressing A33 and A36, and B5 short consensus repeat 4 is critical for this induction.

## Introduction

*Vaccinia virus* (VACV) is a member of the genus *Orthopoxvirus*; it replicates in the cytoplasm (Moss, 2007) and produces multiple distinct virions (Smith *et al.*, 2002; Condit *et al.*, 2006). The first particle formed, intracellular mature virus (IMV), is surrounded by a single lipid membrane (Hollinshead *et al.*, 1999) and represents the majority of progeny virus that remains intracellular until cell lysis. However, some IMV are wrapped by membrane cisternae from early endosomes or the *trans*-Golgi network to form intracellular enveloped virus (IEV). IEV move on microtubules to the cell periphery (Geada *et al.*, 2001; Hollinshead *et al.*, 2001; Rietdorf *et al.*, 2001; Ward & Moss, 2001) and then fuse with the plasma membrane to form a cell-associated enveloped virus (CEV) that remains attached to the cell surface. VACV protein A36 accumulates in the plasma membrane beneath CEV (van Eijl *et al.*, 2000) and is phosphorylated by Src kinases (Frischknecht *et al.*, 1999; Scaplehorn *et al.*, 2002) to induce actin polymerization (Cudmore *et al.*, 1995) that pushes the CEV away from the cell surface. An extracellular enveloped virus (EEV) is formed when CEV is released into the extracellular medium. Viral proteins A36 (Parkinson & Smith, 1994; van Eijl *et al.*, 2000) and F12 (Zhang *et al.*, 2000; van Eijl *et al.*, 2002) are associated with IEV and CEV membranes and are absent from IMV and EEV. In contrast, proteins A33 (Roper *et al.*, 1996), A34 (Duncan & Smith, 1992), B5 (Engelstad *et al.*, 1992; Isaacs *et al.*, 1992), F13 (Blasco & Moss, 1991) and A56 (Payne & Norrby, 1976) are associated with IEV, CEV and EEV. The A56 protein can also form a complex with VACV protein K2 (Turner & Moyer, 2006) and VACV complement control protein (VCP) (DeHaven *et al.*, 2010) and thereby recruit these proteins to the EEV particle (DeHaven *et al.*, 2011). CEV promotes cell-to-cell spread of virus by the induction of actin tails from the cell surface beneath newly synthesized virions, and EEV promotes the dissemination of virus in cultured cells and *in vivo* (Smith *et al.*, 2003).

VACV strain Western Reserve (WR) produces new virions by 5–6 h post-infection (p.i.) and the infectious cycle is complete by 12–15 h (Payne & Kristenson, 1979). Despite this, VACV WR spreads rapidly across susceptible cells at a rate of 1 cell every 1.2 h (Doceul *et al.*, 2010). This rapid spread is not attributable to actin-tail formation from the surface of cells producing new virions (Stokes, 1976; Hiller *et al.*, 1979) or to virus-induced cell motility (Sanderson *et al.*, 1998b; Valderrama *et al.*, 2006) because both of these phenomena are induced at only 5–6 h p.i., too late to explain the rapid spread observed. Furthermore, virus-induced cell motility is a property of only some VACV strains and plaque size does not correlate with induction of cell motility. VACV strain modified virus Ankara (MVA) does not induce cell motility, but can still form large plaques on some cell lines (Drexler *et al.*, 1998; Okeke *et al.*, 2006). Notably, insertion of the *F11L* gene into MVA restores virus-induced cell motility but makes no difference to the plaque size (Zwilling *et al.*, 2010). Therefore, these phenomena cannot explain how VACV spreads so rapidly.

Instead, rapid VACV spread is due to repulsion of superinfecting EEV particles from the surface of cells expressing proteins A33 and A36 (Doceul *et al.*, 2010). These proteins are expressed both early and late during infection (Parkinson & Smith, 1994; Roper *et al.*, 1996), are present on the cell surface (Lorenzo *et al.*, 2000; van Eijl *et al.*, 2000) and form a complex (Röttger *et al.*, 1999; Wolffe *et al.*, 2001; Ward *et al.*, 2003; Perdiguero & Blasco, 2006). Furthermore, VACV lacking either gene spreads slowly, does not make actin tails and forms a small plaque (Parkinson & Smith, 1994; Roper *et al.*, 1998; Sanderson *et al.*, 1998a). A33 and A36 are sufficient for actin-tail formation upon contact with EEV, because addition of EEV, but not IMV or herpes simplex virus type 1 (HSV-1), to cell lines expressing A33 and A36 induced actin-tail formation from the cell surface to repel EEV particles and accelerate spread to uninfected cells. Consequently, it was shown that EEV can bounce or surf across infected cells to reach uninfected cells without the need to replicate in each cell.

To study this phenomenon further we have investigated: (i) whether expression of A33 and A36 from cells increases the rate of VACV spread (size of plaque); (ii) whether the expression of A33 and A36 can inhibit infection by EEV or IMV, and compared this with the effect of proteins A56 and K2, which also form a complex on the cell surface (Turner & Moyer, 2006) and block IMV entry by binding to components of the IMV entry fusion complex (Wagenaar & Moss, 2007; Wagenaar *et al.*, 2008); and (iii) which proteins on the surface of EEV are needed for interaction with the A33–A36 complex to induce actin-tail formation. Data presented show that plaque size is not affected by the expression of A33 and A36 prior to infection, that the A33–A36 complex does not block infection by IMV or EEV to a significant degree, whereas the A56–K2 complex blocks entry of both viruses, and that the EEV protein B5 is needed for the induction of actin tails from the cell surface.

## Results

### Effect of A33 and A36 expression in cells on plaque size

**Fig. 1.**
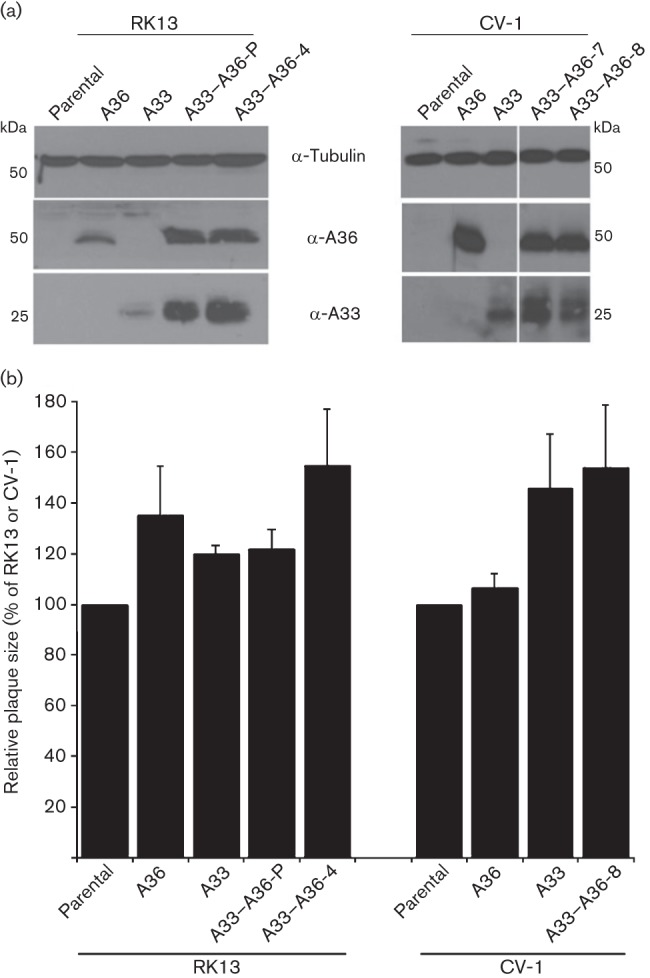
Ectopic expression of A33 and/or A36 does not affect virus spread. (a) Expression of A33 and/or A36 in the RK13 and CV-1 cell lines. Cell extracts were analysed by immunoblotting with antibodies against A33, A36 and tubulin. (b) Expression of A33 and A36 does not affect VACV plaque size in RK13 and CV-1. Cell monolayers were infected with VACV WR for 4 days and the diameter of 12 plaques was measured (see Methods). The relative plaque size is expressed as a percentage of the size obtained for the parental cell line. Data shown are the mean±sd, *n* = 3.

Expression of VACV proteins A33 and A36 early during infection is critical for rapid spread (Doceul *et al.*, 2010) and so we wondered whether VACV would spread faster in cells expressing A33/A36 prior to infection. To address this, VACV plaque size was measured in cell lines that expressed either or both of these proteins. HeLa cells expressing A33 and/or A36 were described (Doceul *et al.*, 2010) but these yielded poor VACV plaques that were not easily measurable. Therefore, we created additional cell lines in RK13 and CV-1 cells that expressed A36-v5 and/or A33–HA using lentivirus vectors as described previously (Doceul *et al.*, 2010) and in Methods. Cell lines expressing A33–HA were created first and then these were transduced with vectors expressing A36-v5 and cloned cell lines were isolated as described previously (Doceul *et al.*, 2010). Protein expression was confirmed by immunoblotting with anti-A36 or anti-A33 antibodies (Fig. 1a). Expression levels in these cell lines was comparable to that achieved in the HeLa cells used previously (Doceul *et al.*, 2010) and was slightly less than obtained in cells infected with VACV in the presence of cytosine arabinoside (to eliminate late gene expression, but which increases early gene expression).

To investigate plaque size, monolayers of these cells were infected with VACV strain WR and plaque size was quantified 4 days later. No difference in plaque size was detected between RK13 cells and those expressing A36, A33 or A36–A33 (Fig. 1b). In CV-1 cells expressing A33, there was a slight increase in plaque size compared with parental cells, but no additional increase when A36 was co-expressed (Fig. 1b). This suggests that the small variation in size of plaques is probably attributable to intrinsic differences in the clonal cell lines rather than expression of A33 and A36. Collectively, these data indicate that spread of VACV was not increased in cells constitutively expressing A33 and A36. However, the plaque size of viruses expressing A33 or A36 under only a late promoter or lacking either gene completely was increased on cells expressing A33 or A36, respectively, showing functional complementation (data not shown).

### Effect of A33–A36 complex on entry of VACV IMV or EEV

**Fig. 2.**
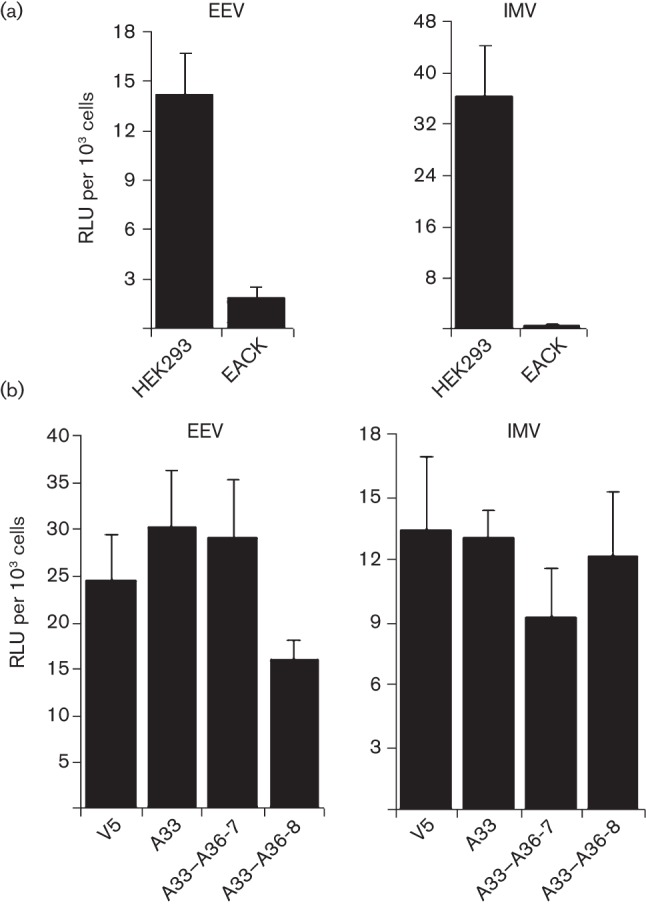
Expression of A33 and A36 does not prevent VACV entry. (a) HEK293 cells and EACK cells (HEK293 cells expressing A56 and K2) were infected with vLuc-WR EEV or IMV. Cells were lysed 1 h after infection and the luciferase activity was determined. (b) The same experiment was performed with the CV-1 v5, A33, A33–A36-7 and A33–A36-8 cell lines. Data are expressed in relative luciferase units (RLU) per 10^3^ cells. The results shown are the mean±sd, *n* = 4, and are representative of three experiments.

It was clear that some EEV added to cells expressing A33–A36 induced actin tails (Doceul *et al.*, 2010) and are repelled to find new cells to infect, but what proportion of EEV enter the cell or are repelled was unclear. To address this, a recombinant VACV (rVACV) expressing luciferase, vLuc-WR, was constructed using established methodology (Mackett *et al.*, 1984). This virus was used to monitor viral entry/early gene expression by measuring luciferase activity shortly after addition of IMV or EEV to cells. Cell line 293EACK13D expressing A56–K2 was also studied because expression of A56–K2 at the cell surface inhibits IMV entry by preventing fusion of virus particles with the plasma membrane (Wagenaar & Moss, 2009). A substantial decrease in luciferase activity was detected after addition of vLuc-WR IMV or EEV to 293EACK13D cells compared with the parental HEK293 cells (Fig. 2a), showing that the A56–K2 complex blocks infection by both IMV and EEV. This result is consistent with both virions having to fuse the IMV membrane with the cell membrane. For EEV this occurs after removal of the outer viral membrane upon contact with glycosaminoglycans on the cell surface (Law *et al.*, 2006). In contrast to the A56–K2 complex, A33–A36 did not reduce IMV or EEV entry to a detectable degree (Fig. 2b). Furthermore, virus cores inside cells were detected by electron microscopy within 30 min of infection with EEV irrespective of whether the A33–A36 complex was present (see Fig. S1, available in JGV Online).

### Proteins on superinfecting EEV particles needed to induce actin-tail formation

**Fig. 3.**
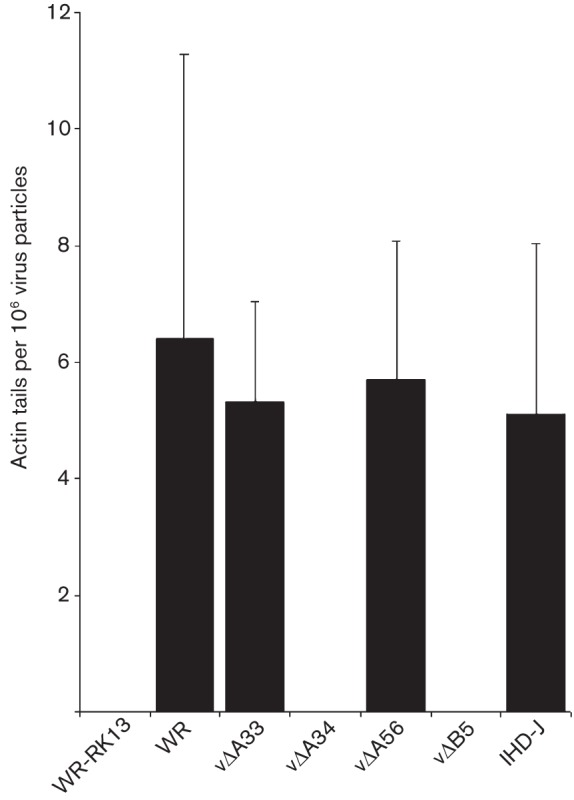
A34 and/or B5 are critical on EEV particles for actin-tail formation. EEV preparations from RK13 cells infected with VACV strain WR, vΔA33, vΔA34, vΔA56, vΔB5, or VACV strain IHD-J were added to the surface of cells at 37 °C for 30 min. Cells were then fixed and stained with phalloidin and anti-F13 mAb to visualize actin tails and EEV particles. Data are expressed as actin tails detected per 10^6^ virus particles and represent the mean±sd, *n* = 3.

The formation of actin tails at the surface of cells expressing A33 and A36 is triggered by EEV but not IMV or HSV-1 (Doceul *et al.*, 2010). Thus, a specific EEV surface molecule(s) must engage the A33–A36 complex leading to actin-tail formation. To determine which EEV surface protein(s) is needed, VACV mutants lacking A33, A34, A56 or B5 individually were tested. Fresh EEV from these mutants was added to cells expressing A33–A36 and the formation of actin tails was measured. The F13 protein is also associated with EEV but is located on the inner surface of the EEV membrane (Husain *et al.*, 2003) and so is unable to bind an extracellular ligand. Consequently, this mutant was excluded from the analysis. These mutant viruses produce different amounts of EEV (Smith *et al.*, 2002) and so it was necessary to compare the number of actin tails produced against the number of EEV particles bound to the cell surface. EEV were quantified by fixing virions bound to cells, permeabilizing the EEV membrane and staining with an anti-F13 mAb (as described in Methods). Data obtained show that EEV lacking A56 and A33 induced actin tails as efficiently as wild-type EEV (Fig. 3). In contrast, EEV lacking A34 or B5 did not induce actin tails.

A34 is a type II membrane glycoprotein that contains a C-type lectin-like domain in its extracellular domain (Duncan & Smith, 1992) and B5 a type I membrane glycoprotein with an extracellular domain composed of four short consensus repeats (SCRs) (Takahashi-Nishimaki *et al.*, 1991; Engelstad *et al.*, 1992; Isaacs *et al.*, 1992). A34 and B5 interact with each other (Röttger *et al.*, 1999; Earley *et al.*, 2008; Perdiguero *et al.*, 2008; Roberts *et al.*, 2009) and A34 is required for the incorporation of B5 in EEV (Earley *et al.*, 2008; Perdiguero *et al.*, 2008; Roberts *et al.*, 2009). Furthermore A34 glycosylation, trafficking and stability depend on B5 (Breiman & Smith, 2010). Given the mutual dependence of A34 and B5, it was impossible to determine from the above data whether A34, B5 or both proteins were required to trigger actin-tail formation. Therefore, mutations in B5 and A34 that influence EEV formation were also investigated.

For A34, a K151E mutation caused enhanced release of EEV (Blasco *et al.*, 1993; McIntosh & Smith, 1996), explaining why VACV strain IHD-J, which contains this mutation, released more EEV than strain WR (Payne, 1979). Using IHD-J EEV, actin-tail formation on cells expressing A33–A36 was normal (Fig. 3). This showed this mutation does not affect actin-tail formation and VACV strain IHD-J, like VACV strains WR and Lister (Doceul *et al.*, 2010), can induce actin tails on cells expressing the A33–A36 proteins.

**Fig. 4.**
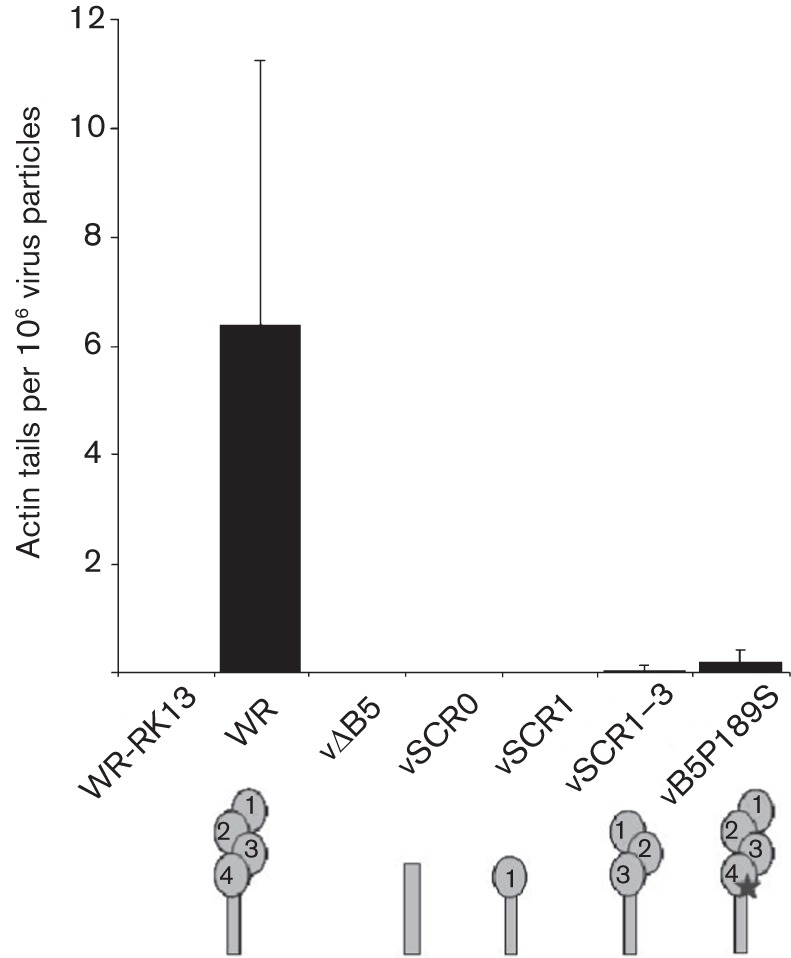
B5 SCR4 is critical for actin-tail formation by superinfecting EEV. EEV preparations from VACV mutants lacking B5 SCR1–4 (vSCR0), 2–4 (vSCR1) or 4 (vSCR1–3) or with a P189S mutation in B5 SCR4 (vB5P189S) were added to RK13 A33–A36-4 cells for 30 min. Cells were then fixed and stained with phalloidin and an anti-F13 mAb to visualize actin tails and EEV particles. The results are expressed in number of actin tails detected per 10^6^ virus particles and represent the mean±sd, *n* = 3.

**Fig. 5.**
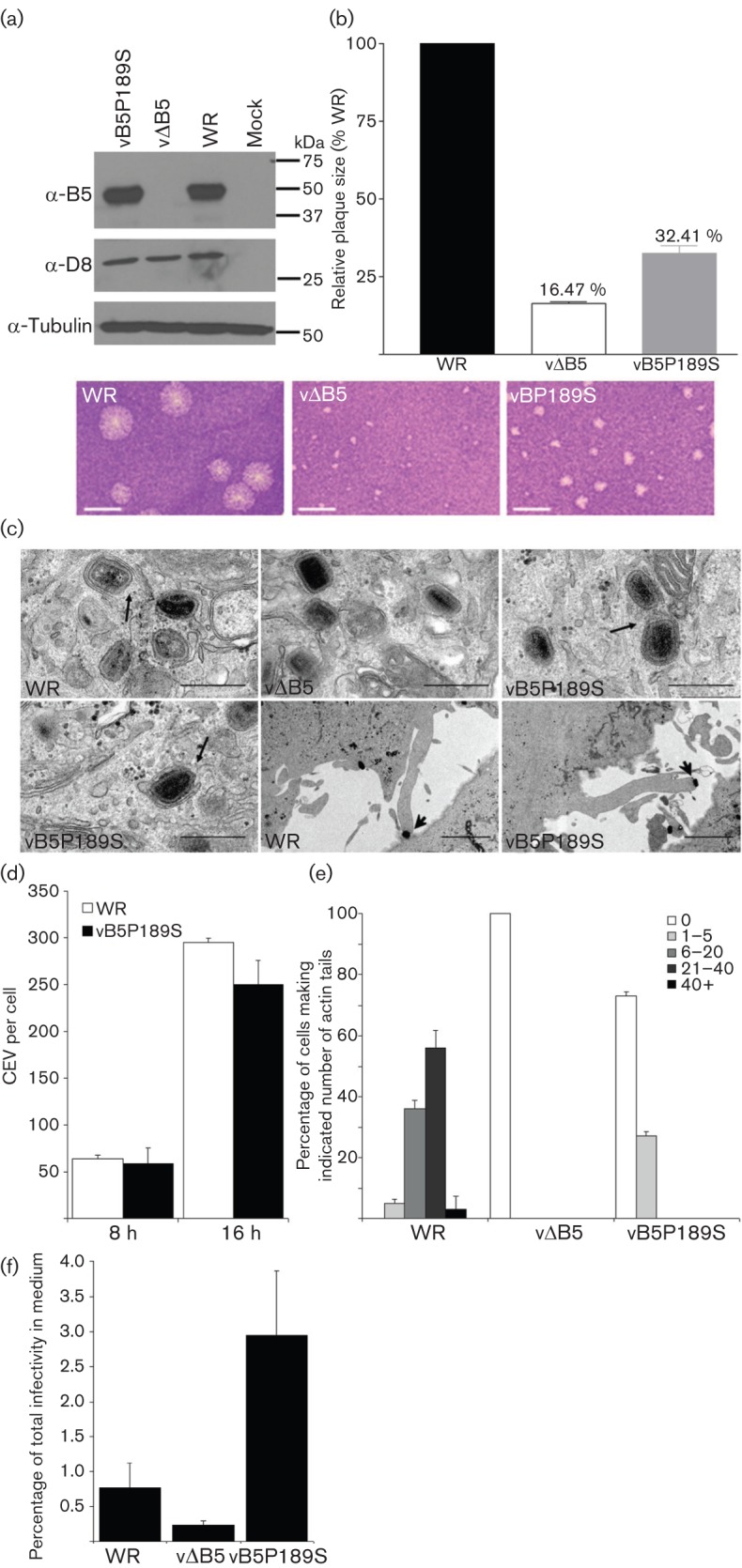
Characterization of rVACV vB5P189S. (a) Immunoblot. Lysates from BSC-1 cells infected with WR, vΔB5 or vB5P189S at 2 p.f.u. per cell were immunoblotted with anti-B5 mAb. (b) vB5P189S forms small plaques. BSC-1 cells were infected with WR, vΔB5 or vB5P189S and plaque size was measured after 3 days. Data are expressed relative to VACV WR and are the mean±sd, *n* = 3. Bars, 2.5 mm. (c) Electron microscopy of HeLa cells infected with WR, vΔB5 and vB5P189S at 2 p.f.u. per cell for 8 h. Black arrows indicate IMV association with wrapping membranes and complete IEV. Arrowhead indicates a virus-tipped actin tail at the surface of cells infected with vB5P189S. Bars, 500 nm (all top row and bottom row left), 2 µm (bottom row middle and right). (d) CEV formation. BSC-1 cells were infected as in (a) and CEV were quantified on cells in 9–10 different fields for each virus. Data shown are the mean±sd of three experiments. (e) Actin-tail production. RK13 cells were infected as in (a) and 16 h p.i. cells were fixed, permeabilized and stained with phalloidin and an anti-D8 mAb. The number of actin tails present at the surface of 50 cells was determined for each virus and classified into five categories: 0, 1–5, 6–20, 21–40 or >40 actin tails. Data shown are the mean±sd, *n* = 2. (f) Extracellular virus formation. RK13 were infected at 5 p.f.u. per cell for 24 h and the number of infectious virions present intracellularly and extracellularly was determined by plaque assay. Data are shown as the percentage of total infectivity that was present in the medium and are the mean±sd, *n* = 3.

Many mutations have been described for B5, including deletion of domains, point mutations and domain swaps with other VACV proteins (Herrera *et al.*, 1998; Lorenzo *et al.*, 1998; Mathew *et al.*, 1998, 1999, 2001; Grosenbach *et al.*, 2000; Newsome *et al.*, 2004; Roberts *et al.*, 2009; Breiman & Smith, 2010; Lorenzo *et al.*, 2012). To determine which SCRs were needed for actin-tail formation, EEV made by mutant viruses lacking one or more SCR was added to cells and actin tails were quantified (Fig. 4). This showed that SCR4 was needed for actin-tail formation. To address this further we studied a P189S mutation within this domain. Katz *et al.* (2002) showed that the P189S mutation caused an increased release of EEV by a virus lacking the A36 protein (Katz *et al.*, 2002) and its introduction into wild-type VACV caused a small-plaque phenotype, increased EEV formation, a failure to induce actin tails from the cell surface of infected cells, and a reduction in virulence (Katz *et al.*, 2003). Later, this mutation was reported to reduce phosphorylation of A36 by src kinases and cause loss of actin-tail formation (Newsome *et al.*, 2004). However, these studies measured actin-tail formation during the exit of newly synthesized virions, rather than during superinfection. To address whether this mutation also affected actin-tail formation during superinfection, a rVACV containing the P189S mutation was constructed (for details see Methods) and the replication and properties of this virus, vB5P189S, were studied (Fig. 5).

### Characterization of rVACV vB5P189S

Immunoblotting showed that the P189S mutation did not affect B5 stability, and analysis of plaque size revealed that vB5P189S formed plaques twice as big as those formed by vΔB5R and one-third the size of plaques formed by VACV WR (Fig. 5a, b). Electron microscopy demonstrated that vB5P189S IMV were formed and wrapped normally to form IEV (Fig. 5c), unlike vΔB5R, which is defective in IMV wrapping and therefore produces little EEV (Engelstad & Smith, 1993; Wolffe *et al.*, 1993). vB5P189S also induced virus-tipped actin projections at the cell surface, although these were fewer than on WR-infected cells. To quantify CEV and actin tails, infected cells were stained with anti-B5 or anti-D8 mAb and phalloidin (Fig. 5d, e). vB5P189S produced similar numbers of CEV at 8 and 16 h p.i. to VACV WR (Fig. 5d), but the number of actin tails were reduced (Fig. 5e) and only 25 % of infected cells showed between one and five actin tails. In contrast, with VACV WR >50 % of cells made >20 actin tails per cell (Fig. 5e). This mutation therefore reduces actin-tail formation substantially but does not eliminate it. Lastly, the release of infectious virus into the medium was measured and data are expressed as the percentage of total infectivity (virus present in cells and culture medium) that was released into the medium (Fig. 5f). This showed that the proportion of total virus represented by extracellular virus was enhanced about fourfold compared with the wild type. These data are broadly in agreement with the study of Katz *et al.* (2003) and the later study from Newsome *et al.* (2004), except that actin-tail formation is not completely inhibited, just reduced significantly.

### B5 P189S reduces actin-tail formation by EEV on cells expressing A33–A36

The ability of vB5P189S EEV to induce actin tails from the surface of cells expressing A33–A36 was then examined. This mutation caused a substantial reduction in actin tails (Fig. 4), but did not eliminate their formation, consistent with the observation on the surface of cells producing new virions (Fig. 5c).

### Incorporation of A34 and B5 into EEV particles of mutant viruses

**Fig. 6.**
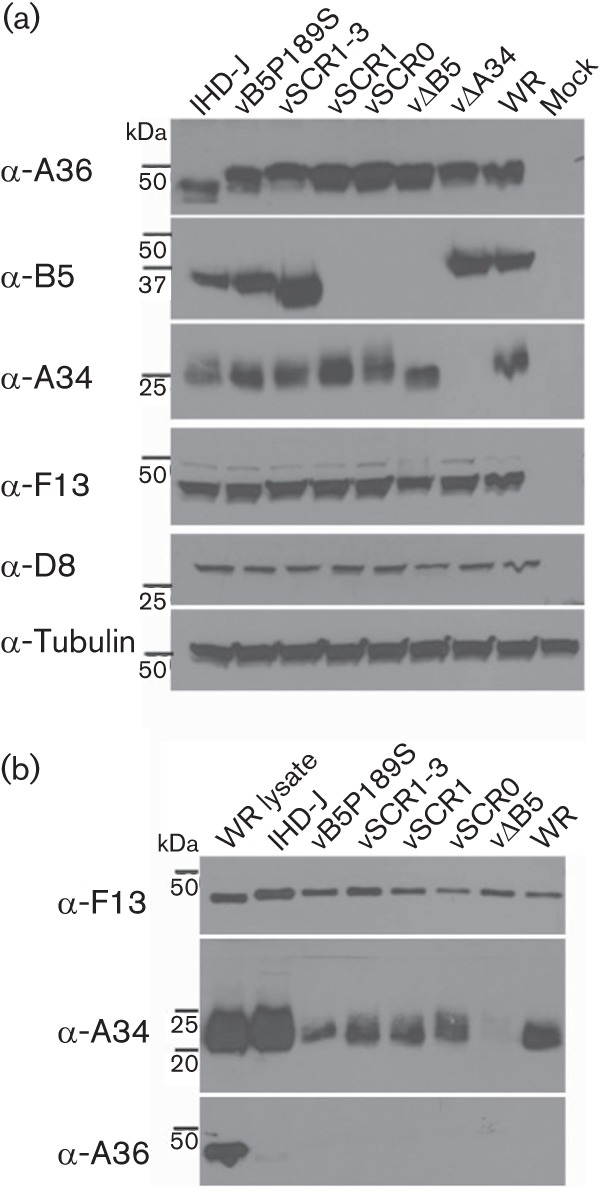
Incorporation of A34 into EEV. (a) Expression of IEV/EEV proteins in cell lysates. HeLa cells were infected at 2 p.f.u. per cell with the indicated viruses for 24 h. Protein lysates were prepared and analysed by immunoblotting using antibodies raised against F13, A36, B5 and A34. An anti-tubulin mAb was included as loading control. Note that B5 was not detected in cells infected with vSCR0 and vSCR1 because the rat mAB 19C2 recognizes B5 SCR2 (Law & Smith, 2001). (b) Incorporation of A34 into EEV. RK13 cells were infected at 3 p.f.u. per cell with the indicated viruses for 16 h. EEV were collected, lysed and analysed by immunoblotting.

The interdependence of A34/B5 for trafficking and incorporation into EEV made it necessary to check incorporation of these proteins into the mutant EEV. Cells infected by each mutant virus expressed the IMV surface protein D8, the IEV protein A36, and the EEV protein F13 at levels comparable to wild-type WR (Fig. 6a). B5 containing SCR2 was detected by mAb to this domain, and A34 was expressed at similar levels by all viruses except vΔA34 (Fig. 6a). Note that the glycosylation profile of A34 was different in cells infected with this deletion virus (Fig. 6a) as reported previously (Breiman & Smith, 2010). In EEV particles, A34 was present in WR and IHD-J and also the mutants lacking B5 SCRs (Fig. 6b), consistent with another study (Perdiguero *et al.*, 2008) showing that A34 and B5 can interact through the SCRs and the C-terminal region of B5. B5P189S was also incorporated into EEV, consistent with this mutation not affecting B5–A34 interaction (Perdiguero *et al.*, 2008). Collectively, these data indicate that the impaired ability of vSCR0, vSCR1, vSCR1–3 and vBP189S EEV to induce actin tails upon addition to cells expressing A33–A36 is caused by the deletion or mutation of B5 SCR4. These data also indicate that CEV-mediated induction of actin tails from the cell surface during virus exit, and actin-tail formation from the surface of cells binding superinfecting EEV each require protein B5.

## Discussion

The rapid spread of VACV from cell to cell requires early expression of proteins A33 and A36 on the cell surface, contact of this complex by a CEV/EEV and polymerization of actin to drive superinfecting EEV away towards uninfected cells. In this paper we have investigated additional features of this phenomenon and addressed the following questions: (i) does the constitutive expression of A33–A36 in cell lines make VACV spread faster than it does in normal cells?; (ii) does this complex prevent the cells being infected by the majority of EEV particles?; and (iii) which components of the EEV particle are needed for the interaction with the A33–A36 complex to induce actin polymerization?

Early expression of A33–A36 is crucial for rapid spread, and so, if these proteins were already present on cells before infection, the spread might be accelerated because there would be no delay between infection and when these proteins are present at sufficient level to induce actin tails after contact with superinfecting EEV. However, measurement of plaque size in cells expressing A33–A36 showed no increase over controls. While these data do not show an increase under the conditions tested, the optimal expression level and ratio of A33/A36 remain unknown and it is possible that cells expressing higher levels might be better at inducing actin tails upon addition of EEV. It is also possible that although the A33–A36 complex is sufficient for induction of actin polymerization by EEV, there could be other virus proteins that influence the efficiency of this process, perhaps by stabilizing the complex.

To investigate whether the A33–A36 complex influenced virus entry, an rVACV expressing luciferase under an early promoter was used. Measurement of luciferase expression early after infection with IMV or EEV showed no reduction in cells expressing the A33–A36 complex compared to parental cells. In addition electron microscopy showed virus cores inside the cytosol shortly after infection. Even though the majority of EEV enter cells expressing the A33–A36 complex, the A33–A36 complex is important for rapid spread, and viruses lacking either gene or expressing either gene only late during infection form small plaques (Parkinson & Smith, 1994; Roper *et al.*, 1998; Doceul *et al.*, 2010). Evidently, the rapid spread to uninfected cells could be achieved by only a small percentage of total EEV. In comparison, a cell line expressing the A56–K2 complex (kindly provided by B. Moss, NIH, Bethesda, MD, USA) that binds the IMV entry fusion complex (Wagenaar & Moss, 2009) was also studied. Consistent with previous reports, this cell line blocked infection by IMV, and we show here that it also blocks infection by EEV. The latter result is logical because after an EEV particle has lost its outer envelope either after contacting glycosaminoglycans (GAGs) on the cell surface (Law *et al.*, 2006) or following endocytosis and acidification (Schmidt *et al.*, 2011), the IMV particle must still fuse with the cell membrane and the presence of the A56–K2 complex would block this. Deletion of either K2 or A56 induces a fusogenic plaque phenotype but the plaque size is similar to the wild type, indicating this does not impact on virus spread (Law & Smith, 1992; Zhou *et al.*, 1992; Law *et al.*, 2002). These data indicate that it is the A56–K2 complex that prevents superinfection, and the function of the A33–A36 complex is to promote rapid spread.

To address which EEV protein is needed to engage the A33–A36 complex, mutants lacking EEV proteins were tested for actin polymerization on A33–A36 cells. This showed that A33 and A56 were not required, but A34 and B5 were. Given that the A34 protein is needed for efficient incorporation of B5 into EEV (Earley *et al.*, 2008; Perdiguero *et al.*, 2008; Roberts *et al.*, 2009), the phenotype of the vΔA34 EEV could be due to loss of B5. Analysis of additional mutants was consistent with this proposal, and SCR4 of B5 was implicated. This was also supported by analysis of a B5 protein bearing a P189S mutation in SCR4 which had a defect in actin-tail polymerization. B5 and A34 were incorporated into EEV particles of these mutants and so the defect was not attributable to lack of A34. Previously it was shown that the P189S mutant was not able to induce actin-tail formation at the surface of cells producing new virions and that B5 SCR4 was required for actin polymerization (Katz *et al.*, 2003; Newsome *et al.*, 2004). Our data on the induction of actin tails during release by the P189S mutant and during superinfection are broadly in agreement with these reports, although the defect in actin-tail formation is not absolute either during virus exit or during superinfection. It is notable that the A34–B5 complex is not only important for actin-tail induction by CEV or EEV, but it is also important for the disruption of the EEV envelope upon contact with GAGs on the cell surface (Law *et al.*, 2006; Roberts *et al.*, 2009).

Most of the A36 protein is situated in the cytoplasm, whereas A33 has most of its polypeptide outside the cell. So it is likely that A33 acts extracellularly to recognize EEV via B5 SCR4 at the cell surface and A36 is then phosphorylated intracellularly to initiate actin polymerization. A33 and B5 interact with each other but this interaction requires the transmembrane region of B5 (Perdiguero & Blasco, 2006). Data presented here suggest that B5 and A33 might interact even when anchored in different membranes, possibly via the extracellular domains, although direct binding data are needed to confirm this. Interestingly, the X-ray crystal structure of the A33 ectodomain has revealed that A33 contains an unusual C-type lectin like domain (CTLD) that is probably involved in binding ligands (Su *et al.*, 2010).

Lastly, the involvement of A33 in rapid virus spread may help explain other observations. First, antibodies to A33 can help provide protection against orthopoxvirus infection, without neutralizing the EEV particle (Galmiche *et al.*, 1999; Fogg *et al.*, 2004). Secondly, VACV plaque formation is not inhibited by polyclonal antibody raised against a VACV infection, so VACV can spread from cell to cell in an antibody-resistant manner; but if the A33 protein is absent VACV spread is prevented by antibody (Law *et al.*, 2002). The requirement of A33 to facilitate rapid spread of virus may explain both observations, because binding of antibody to A33 on the cell surface A33 could block spread and therefore reduce the induction of disease. Further, rapid spread of VACV requiring the A33–A36 proteins may explain why spread is normally resistant to antibody and therefore why it becomes sensitive when the A33 protein is absent.

In conclusion, this study provides insight into the mechanism evolved by VACV to repulse superinfecting virions and enhance cell-to-cell spread of the virus. A better understanding of these mechanisms could identify viral targets and lead to the discovery of new viral drugs such as molecules designed to neutralize B5 and A33.

## Methods

### 

#### Cells and viruses.

BSC-1, HeLa, CV-1 and RK13 cells were grown as described previously (Kerr *et al.*, 1991; Mathew *et al.*, 1999). HEK293 cells expressing A56 and K2 (EACK cells) were described by Wagenaar & Moss (2009). The VACV strains Western Reserve (WR) and International Health Department (IHD)-J (Alcamí & Smith, 1992) and the deletion mutants vΔA33R (Roper *et al.*, 1998), vΔA34R (McIntosh & Smith, 1996), vΔB5R (Engelstad & Smith, 1993) and vΔA56R (Sanderson *et al.*, 1998a) were used. Viruses lacking B5 SCR2–4 (vSCR1), SCR4 (vSCR1–3) or all SCRs (vSCR0) were as described earlier (Herrera *et al.*, 1998; Mathew *et al.*, 1998). VACV was titrated by plaque assay on BSC-1 cells as described previously (Law *et al.*, 2002).

#### Generation of HeLa, RK13 and CV-1 cells expressing A33 and/or A36.

A lentivirus vector derived from pdlNot’MCS’R’PK and expressing A36-v5 was described earlier (Doceul *et al.*, 2010). A DNA fragment encoding A33–HA was amplified from pcDNA3-A33 with oligonucleotides A33RHA-forward (5′-CGCGGATCCCACCATGATGACACCAGAAAAC-3′) and A33RHA-reverse (5′-GGAATTCCATATGTTAAGCGTAATCTGGAACATCGTATGGGTAGTTCATTGTTTTAACAC-3′) containing *Bam*HI or *Nde*I sites (underlined) and a haemagglutinin (HA) tag. The PCR product was digested with *Bam*HI and *Nde*I and ligated into lentivirus vector pdlSurPkIB that was derived from pHR-SIN-CSGW (Demaison *et al.*, 2002) and restricted with *Bam*HI or *Nde*I. Control lentiviruses obtained from pdlNot’MCS’R’PK and lentiviruses expressing A33–HA or A36-v5 were produced as described previously (Demaison *et al.*, 2002). RK13 and CV-1 cells were infected with these lentiviruses and selected using blasticidin (pdlSurPkIB-derived lentiviruses) and/or puromycin (pdlNot’MCS’R’PK-derived lentiviruses). CV-1 v5 cells were produced after infection with control lentiviruses. Clonal cell lines expressing A33-HA or A36-v5 in RK13 (named RK13 A33 and A36, respectively) and CV-1 (named CV-1 A33 and A36, respectively) were isolated subsequently to optimize expression of the viral proteins. Clonal CV-1 and RK13 lines expressing A33–HA were transduced with lentiviruses expressing A36-v5 to create CV-1 and RK13 A33–A36-P cells. Single clones were then isolated to obtain CV-1 A33–A36-7, CV-1 A33–A36-8 and RK13 A33–A36-4. HeLa v5 and A33–A36 were generated as previously (Doceul *et al.*, 2010).

#### Generation of VACV expressing luciferase (vLuc-WR).

An rVACV expressing luciferase was generated by insertion of the firefly luciferase gene from pGL3 (Promega) into pGS20 (Smith *et al.*, 1983) downstream of the p7.5K early/late promoter forming pGS20-Luc. pGS20-Luc was transfected in CV-1 cells infected with VACV WR and an rVACV was isolated as described previously (Mackett *et al.*, 1984). The presence of the luciferase gene within the thymidine kinase (TK) locus of vLuc-WR was confirmed by PCR (Fig. S2).

#### Generation of rVACV vB5-P189S.

A DNA fragment containing the *B5R* gene and 429 bp upstream and 636 bp downstream was cloned into pSJH7 (Hughes *et al.*, 1991). A P189S mutation was introduced by site-directed mutagenesis using primers B5P189S-forward (5′-GTCAACAAAAATGTGATATGTCGTCTCTATC-3′) and B5P189S-reverse (5′-GATAGAGACGACATATCACATTTTTGTTGAC-3′) forming pB5R-P189S which was transfected into cells infected with vΔB5R (Engelstad & Smith, 1993). The resulting rVACV, vB5P189S, was selected by its increased plaque size compared with vΔB5R and its fidelity and purity were confirmed by PCR and sequencing.

#### Virus entry assay.

vLuc-WR stocks were grown in TK^−^143 cells and cytoplasmic lysates were used as IMV. EEV was prepared from the supernatant of vLuc-WR-infected RK13 cells (3 p.f.u. per cell, 20 h) by centrifugation (2000 ***g*****, 10 min) to remove cell debris. vLuc-WR EEV or IMV were used to infect cells at 1 p.f.u. per cell for 2 h at 4 °C. Unbound virus was removed, cells were incubated at 37 °C for 1 h, cell lysates were prepared in Cell Lysis Buffer (Promega) and luciferase activity was measured.

#### Plaque-size measurement.

The diameter of plaques (*n* = 12) formed by VACV WR was determined as described by Law *et al*. (2002) in three independent experiments using AxioVision Rel. 4.6 software (Zeiss).

#### Actin tail and CEV quantification.

Cells were infected (2 p.f.u. per cell, 8 or 16 h), and stained for actin, D8, F13 or B5 to quantify actin tails or CEV, respectively, as described by Herrero-Martínez *et al.* (2005). Cells were permeabilized with Triton X-100 (VWR) when required, blocked in 0.5 % BSA and incubated with rat anti-F13 mAb (15B6; Schmelz *et al.*, 1994), rat anti-B5 mAb (19C2; Schmelz *et al.*, 1994) or mouse anti-D8 mAb (AB1.1; Parkinson & Smith, 1994). Secondary Alexa 488- or Alexa 546-conjugated donkey anti-mouse or anti-rat were used to detect bound primary antibody. Actin was visualized with phalloidin labelled with Alexa Fluor 488 or 546 (Molecular Probes). Samples were mounted in Mowiol–DAPI mounting medium. Microscopy was carried out with a Zeiss 510 Meta confocal microscope (Zeiss).

#### Spinoculation of EEV and quantification of actin tails.

Fresh EEV were spinoculated onto cells and EEV and actin tails were quantified as described previously (Doceul *et al.*, 2010). The number of cells per coverslip was determined using a Countess automated cell counter (Invitrogen) (*n* = 2) and the number of bound EGFP-positive virions present per cell was counted in five different fields.

#### Electron microscopy.

Infected cells were processed as described previously (Doceul *et al.*, 2010) and collected using analysis version docu software (Olympus Soft Imaging Solutions).

#### Immunoblotting.

Immunoblotting of cell lysates (Doceul *et al.*, 2010) or EEV (Law *et al.*, 2006) was performed as described previously. Antibodies used were anti-A33 mouse mAb (A33-1), rabbit anti-A36 antibody (Röttger *et al.*, 1999), rat anti-B5 mAb 19C2 (Schmelz *et al.*, 1994), mouse anti-D8 mAb AB1.1 (Parkinson & Smith, 1994), mouse anti-A34 mAb 34-1 or mouse anti-α-tubulin mAb (clone DM1A, Millipore). Bound primary antibodies were detected by HRP-conjugated anti-rabbit (Stratech Scientific), anti-mouse (Stratech Scientific) and anti-rat (GE Healthcare) antibodies.
